# On Some Ways of Manipulating the Filling Materials

**Published:** 1893-09

**Authors:** 

**Affiliations:** London, Eng.


THE
AMERICAN JOURNAL
—OF—
### DENTAL SCIENCE.

VOL. XXVII. BALTIMORE, SEPTEMBER, 1893. No. 5.

ARTICLE I.

### ON SOME WAYS OF MANIPULATING THE FILLING MATERIALS.

BY MR. HALLIDAY, LONDON, ENG.

*Mr. President and Gentlemen :—*

When I decided to write a paper on this subject, I did not anticipate the task I had set myself. The subject is about the most interesting for us all that we can have, and once having started on it, it is difficult to stop. As a matter of fact, a separate paper could well have been written on any and each one of the divisions alone, but I found that out too late. I must therefore ask you to be patient with me through a long paper, the statements made by one authority being contradicted often by another, and I have often put in both so that you may signify in the discussion the method you consider the best.

### GOLD.

Non-cohesive gold is retained in position by mutual interlocking of the pieces composing the plug, as well as keyed in by last portions introduced. It may be introduced in the form of cylinders, rope or tape. If cylinders are used they are packed like cigars in a tumbler, each cylinder standing on end in the cavity, with the end projecting from the cavity mouth. They are always compressed laterally against the walls, and as the cavity gets full, well pressed into the undercuts. When nearly full a sharp wedge-shaped instrument is introduced into the middle of the mass and worked sideways so as to leave an opening which should leave access to the middle of the cavity. Into this opening another cylinder should be forced, even compressed by rolling in fingers before putting in. When the cavity will take no more cylinders, tape may be wedged in. Tape may be used by cutting into strips of the width required and placing several cross ways in the form of a star, and placing this star on the floor of the cavity tucking into the undercuts. Several stars are then inserted in this way, and afterwards the ends are tucked in, not at the circumference, by a fine-pointed instrument. In whatever way tape is used for this, it consists entirely in placing the several pieces so that they may be interlaced by successive folding one piece on another. This form of gold filling when the cavity is full and the surface is condensed is cut down to the bite and burnished, for however well the fold may be condensed over the edges, burnishing will improve the edges; cavities are, therefore, more advantageous for this method if the edges are bevelled, so that the gold may be well burnished over them.

To obtain the best results with cohesive gold it is necessary to pack it in small pieces. It may be used in the form of crystal and sponge gold and foil, this last being used either folded into tape, rolled into rope, or made into cylinders.

When used as tape, it is generally used with a mallet. The gold may be started by points, drilled into the dentine or having the floor entirely, or partly filled with cylinders, being filled with cylinders just enough to give a firm start for the mallet. This is less painful than starting points. When retaining points are used the gold should be packed in each, taking care to well condense it, a little above the floor of the cavity, a piece of tape is then worked on the first one, and then the other, making a bridge between them, on this more is built, fully getting it into the undercuts, and over the cervical edge. This firm foundation may be made either by using the automatic mallet when the starting points are filled, or by hand pressure. It may then be continued either by hand pressure or by mallet. It is important in tape filling by the mallet that the tape be evenly folded, as, should it get crumbled up in a lump, it may not condense properly or not even cohere to the last layer if the foil is very heavy. If it goes down well under the plugger, all is right, the only thing then being that after the filling has been in a few months the surface will show spotty. The tape shall be burnished over the edges with the plugger point.

Malleting should not be continued on any piece of gold till it gives a clear sound, it should always be a dull sound, for when you get a clear sound from the malleted gold it shows that the cohesion is getting impaired, and the gold become brittle and very liable to curl away from the edges. Gold for hand pressure may be either rope, tape or cylinder. The filling is started by taking a cylinder in the bottom of the cavity in one corner, and condensing it there, then holding it in position, another on the end of this, and then another, holding the enlarged mass until you have packed enough in the base of the cavity to stay in by itself. It may be started by taking a large cylinder, annealing it, and condensing it at the ends first. If it is condensed in the middle before the ends are thoroughly condensed, it is pretty sure to curl up. The cavity should then be built up, either with cylinders, added one by one, each being carefully condensed, or by tape, the end of the first piece being made to adhere to the cylinder mass by hand pressure and then condensed by the mallet. Cylinders, especially if at all large, should be condensed first at the ends, and the middle last as if the middle is done first, in condensing the ends the whole piece is likely to tear away, as the gold has a great tendency to form into a ball under the plugger. Cylinders should always be laid sideways on one another so as to get the layers parallel, a better cohesion resulting in this way, and also giving a better face to the filling. A cylinder-built filling is as good as a tape, but does not give such a hard surface nor take such a high polish.

A good way to start a cavity when the cylinders give trouble, is by balling up, and also in cavities rather difficult of access is to take an unannealed cohesive cylinder and condense it in a cavity, flooring the whole cavity with this, and then working annealed cylinders all around the edges of the cavity and well into all undercuts. This gives a nice firm floor. Cylinders do not need a heavy pressure, a slight pressure accompanied by a slight rocking motion of plugger is more efficient. Rope is a very good way of using gold by hand pressure. A whole sheet is rolled up and cut into fair-sized pieces, and if the cavity is fairly accessible, a large foot-plugger may be used and the gold quickly massed in. This form has one good point—sometimes, when for some reason the gold fails to stick, and neither tape nor cylinders go nicely, this form will cohere very well. Another good point is the very much lessened possibility of burning the gold in annealing. The only thing in packing gold in this way is to be extra careful of the edges, for the gold packs so easily.

If cylinders are too much annealed they are very likely to curl up, if not enough they may cohere, but the filling is not so solid.

Cohesive gold may be packed by nearly or quite smooth pluggers in foil or cylinders.

With regard to plugger points. If they are serrated the teeth should be very fine. Some contend that such a perfect filling cannot be made with serrated points as with smooth ones, and that the balling up and curling away of gold from the edges is to be ascribed to the use of serrated pluggers, and that the face of the instrument must be smooth to allow of lateral expansion of foil. Dr. Thompson recommends all foil to be non-cohesive and annealed as wanted, as when annealed only once it is very cohesive and very soft. If annealed more than once it is very hardy and unruly.

Foil may be folded three or four or more times according to the liking of the operator, or rolling it into a rope should be done in a napkin, as the moisture from the fingers is apt to spoil the surface of the gold. The gold may be crumpled by laying a sheet on a velvet covered pad, and drawing a similar pad lightly over it.

Many porous fillings are the result of too rapid packing and using too thick gold, and again, from too long continued malleting, which causes gold to lose much of its cohesiveness; instead of packing dead against walls it is driven from them by the excessive vibratory motion which partially destroys the close contact obtained before. Light and prolonged tapping with small instruments will better unite the particles of the gold, and more readily adapt it to the cavity walls than a bigger instrument and a heavy blow. Delicate, light and rapid manipulation is best. On the other hand, Dr. Leslie and others contend that as large a point as possible should be used, as few blows as possible to condense it, and though small points must be used sometimes round the edges, large points will give better cohesion. He says, "The blacksmith uses a hammer with as large a face as possible when welding iron, and would laugh at any one who should tell him he would make a better weld the smaller the face of the hammer."

Crystal gold should be used in small pellets or strips and pressed quite gently all over with a large pointed plugger so as to condense each individual pellet, then increase the pressure. After the foundation of the filling thus made, they should be condensed in the usual way with mallet or hand pressure. Do this with each pellet. If filled entirely with hand pressure the last layer should be malleted and then burnished, as the burnisher spreads the gold well over the edges. Sponge gold should only be used with very firm cavity walls, and in very small pieces, as the pellets are liable to be condensed on the surface and not underneath, making a porous filling.

Cavities may be lined with non-cohesive gold by first placing a layer of non-cohesive gold at the cervical edge and covering it with cohesive, well filling in the corners. Then place a soft cylinder against one wall joining the first layer, and a cohesive one on the top, making it adhere to the first layer of cohesive. Keep on adding all round a soft cylinder against the wall and a cohesive one to keep it there, working each cohesive one into the last. When the whole of the cavity except the floor is filled, place a soft cylinder as large as possible on the floor, and condense lightly and cover with cohesive foil and work this on to the cohesive lining round and then finish with cohesive. By this means great pressure on frail walls is avoided. They may also be lined by folding soft foil into a heavy weight and laying against the walls and the floor and the cervical edge, tucking into the undercuts and fixing with cohesive, allowing the lining to project beyond all the edges till the cavity is well filled with cohesive.

When a filling has been started with non-cohesive and is to be finished with hard gold, in starting the hard gold, tape may be made to adhere fairly well to a soft gold if well worked into it by a serrated plugger, or cylinders may be condensed into corners and undercuts and held there, while more is added, till a firm base is made and it is then finished in the usual way.

Cavities may be started by putting a floor of osteo in the cavity, and before it is quite set embedding a condensed cylinder in it. When the osteo is properly set the mallet may be used on it at once.

The Herbst method of packing sofc gold by rotating burnishers is said to make a hard, solid, and more perfectly adapted filling with greater ease and in less time than in any other way. For this method there must be four walls to the cavity, either natural or made with a matrix. The cavity must be bigger inside than at the mouth, but not necessarily undercut, and no retaining points are used. The enamel edges must be square. The cavity is then loosely filled with large unannealed Wolrab's cylinders. A polished steel burnisher, pear-shaped, as large as the mouth of the cavity will permit, is introduced and slowly revolved by the engine, while by firm, steady pressure the gold is squeezed to the sides and edges of the cavity until it is thoroughly condensed and presents a highly polished non-cohesive surface. A blunt-pointed instrument is then substituted for the pear-shaped burnisher, and the surface is prodded all over, the engine being rotated more rapidly and moderate pressure being used. The gold is thus still more thoroughly condensed; it loses its hard polished surface and becomes a hard solid mass of cohesive gold. This is repeated until the cavity is full and the matrix is removed, the filling cut down to the bite and polished. When the burnishers become coated with *au*, they should be cleaned on a piece of block tin or fine glass paper. If the instrument is kept too long in one place a painful amount of heat is generated. The matrix should be well beyond margins of the cavity in every direction. In the front teeth the tooth to be operated on and the two or three adjoining should be embedded in shellac, with a platinum lining for a matrix. If two interstitial cavities are to be filled they may be filled together and afterwards separated by drilling, passing a needle several times through the filling and then using a saw.

If cohesiveness is temporarily lost a fine cut bur over the surface of the gold will restore cohesion, or going over the surface with a finely serrated plugger to remove the polished surface.

If the surface of a gold filling should become accidentally swamped during filling, dry as much as possible by ordinary means and then take a burnisher which has got gilded and rapidly rotate it for a short time over the filling, and sufficient heat will be generated to make it dry and cohesive. Very hard fillings can be made this way that will stand the ink test.

As changing the polishing bur for a dull blunt bur took up a lot of time, hand instruments with round polished heads are now used for the first condensing, being followed by engine, the hand instruments being rotated ½ or ¼ turn.

The last layer can be condensed with the mallet if it is liked. Agate burnishers do not get gilded so quickly, and so save the time occupied in cleaning. When the burnishers get gilded they are apt to tear the gold and also generate far more heat than when gilding has not taken place.

The matrix for front teeth may be made of watch spring, sloped away from the cavity so that the slope of the matrix heads right into the cavity, causing all the force of the rotating burnisher to be expended in driving the gold against the walls of the cavity and spring, and not forcing it between the teeth.

Cavities may be filled this way with tin and finished with gold. The tin should be fresh. The last layer of tin to which the gold is to be attached must be very carefully condensed and tried all over, especially at the margins, to see that it is thoroughly condensed, finally trimming away the excess with an excavator. The last layer should be completed with partially annealed gold, packed either by hand pressure, or mallet, or hand, or engine burnishers rotating, but hand rotation is best. This should be still further condensed by the engine burnisher. The gold under the rotating instrument readily unites with the tin, the gold being heated and condensed by hand, which it does not do with the mallet or hand pressure alone without rotation. The filling should then be cut down to bite and polished. This can be done in crown cavity of molar or in an interstitial cavity with a matrix. The walls for this need not be so strong as for gold, and this is thought by Herbst to be better than esteo or amalgam.

When there is a difficulty in making the first layer go in its place, press a few slightly heated cylinders into the floor of the cavity and then fill the cavity two-thirds or quite fully, of clean dry cotton wool, and burnish. The gold will then be found to have gone down beautifully and uniformly. Less gold should be put in at a time when it is condensed under cotton. For the second layer slightly heated cylinders are placed on the gold already condensed, pressed into position and then burnished through cotton as before; then the wool is removed and condensation is accomplished without wool. Examine for soft places and make good with small cylinders. The advantages of the wool are that it makes the gold secure on the floor of the cavity, and is equally condensed all through, and not harder on the surface, because the gold does not come into contact with the instrument. It may be made quite hard and solid after the wool is removed by pressing revolving instrument over the surface. The cavity may be lined with gold by taking a piece of heavy foil suitable to the size of the cavity, or a condensed cylinder, placing it in position and pressing firmly against the tooth with cotton wood; the lining fairly fixed in position, the cavity is filled two-thirds with wool, strong pressure being used by rotating burnisher to force gold under the wool against the walls —then roll a piece of tin into a ball to fit the cavity and burnish through this especially on to the margin. This makes gold quite non-cohesive, which does not matter as only a gold lining is wanted. When tin is removed every undercut, etc., made for retaining filling will be clearly imprinted on as if stamped in gold, so accurately is it pressed by the tin. Before the amalgam is inserted, the gold must be varnished with copal or other varnish, which must be thoroughly dried by hot air before the amalgam is added. The amalgam is packed by hand in ordinary way, not by rotation. Slope filling towards back to show as little amalgam as possible.

In finishing off gold fillings in crown cavities the gold may be cut down to the bite and free from the edges, leaving nowhere overhanging edges of gold, either by fine cut burs or conundrum wheels or points, and then finishing with fine stones and pumice on wood or buff points.

Non-cohesive gold should not be polished, always burnished, as however well it may be packed, burnishing will improve the edge.

Interstitial cavities may be trimmed first of all with a file, if accessible, as a dividing file, and then shaped with emery discs, and the thickened rim discs for the cervical edge when it is not wanted to cut away too near the crown, and in contour fillings. If the discs are soaped with a little hard dry soap they cut better, and are less liable to crumple if caught against another tooth; another good point about the soaped discs is that all the gold cut from the filling adheres to the discs, and can be saved instead of wasted, as is the case with dry discs. When by the coarse discs the filling is made flush everywhere with the edges, finer discs are run to take out scratches, and finer still, and if they are all soaped, the very fine one, if a trifle damp after soaping, will give a bright polish, which, in a case where there is no room to run a rubber disc, is very convenient.

Amalgam fillings, to secure the best results should also be treated this way, as food being collected by overhanging edges is a great source of re-decay. Of course, stones used for amalgam are best kept away from gold.

Amalgam, in addition to polishing, may be burnished, but always up and down a filling, not across, as this gives it a whiter look, and polished amalgams retain their color longer and better than if not polished.

### TIN.

Tin is packed in the same way as non-cohesive gold, but is easier, softer and quicker to work. It may be introduced by the Herbst method, the tin going down beautifully under the burnisher, and seeming to cohere somewhat, but for this the tin must be fresh with a bright surface. Tin being soft is not much good on a masticating surface, and should always be protected with a layer of gold.

In a crown cavity, if it be started with tin, it should be stopped at the juncture of the enamel and dentine, and there have the gold built on it. In an interstitial cavity it may be packed as high as liked, always providing sufficient hold is left to make the superposed gold perfectly firm.

Tin and gold used together, one sheet of tin between two of gold, non-cohesive, is used as rope or tape and packed non-cohesively, and makes a splendidly hard filling.

The presence of moisture is an advantage rather than a defect in using this, as it causes the change, whatever it is, to take place sooner than if it was dry. It can be safely used in cavities which were unable to be perfectly excavated or sterilized and if liked, the first few pieces may be dipped in carbolic acid or oil of cloves if the filling is near the pulp and the cavity rather sensitive.

Interstitial cavities may be entirely filled with this or have top part filled with gold. In crown cavities it soon gets hard enough to withstand mastication.

Where the discoloration is likely to show, the walls may be lined with gold if desired.

It is finished, like non-cohesive gold, by being burnished, not polished.

### AMALGAMS.

On the question of amalgams there is a very great diversity of opinion among the many experimenters. This holds both as to the properties and methods of working.

Copper amalgam requires to be heated till bubbles of mercury cease to appear, when it is crushed in a mortar and vigorously pounded till quite soft and plastic. In this condition it is usually too soft to use, as it would contract very much and curl away from the edges. If squeezed hard between the fingers some mercury will be squeezed out. The floor of the filling may be made of this, and then have enveloped the rest of the amalgam in a piece of chamois skin and squeezed out all the mercury possible with a pair of pliers, finish the filling with this. In this way there is less contraction than if it had been all of the same consistency.

When the cavity is near the pulp, and there is not room to put a floor of osteo, the cavity may be painted with a thick chlora percha, and the amalgam introduced before the solvent has all evaporated, else hard biting may cause pain if the filling rests on a foundation of gutta-percha.

A layer of osteo may be placed on floor and the amalgem added before it is quite set, giving extra hold where the retention power of cavity is weak.

For a non-conducting floor for amalgam (or for esteo), a thin piece of cork dipped in carbolized resin is very good, as it is sedasive and antiseptic and adhesive, beside being a non-conductor of heat.

All platinum amalgams should have as little mercury as possible, and must be worked very quickly and in such a manner that they are not liable to be disturbed till hard. The platinum combines rapidly and strongly with the mercury, but its power of setting lasts but a very short time.

Gold fillings which have failed at the cervical edge may be patched with amalgam; if the amalgam does not hold well, a little mercury rubbed on the edge of the gold will make it unite readily.

There are two points about amalgam fillings that are important; the first is that wherever there is a bevelled edge of enamel, it does not matter what amalgam is used, or how it is inserted, at that point the filling will leak. This remark applies equally to gutta-percha. The second point is that amalgam, soft or hard, does not make a good filling if it is simply placed in the cavity and tucked into the undercuts and then trimmed.

Mr. Fletcher says that a small ball-headed instrument is an absolute necessity in order to obtain sound plugs which do not discolor the dentine. He says, "there is one way in which really first rate fillings can be made with second rate amalgams, and which gives such special results in the theoretical testing as to enable a practiced hand to make with a good amalgam the most absolutely faultless work. This is to take a small portion of the amalgam, anchor it in some corner or retaining point, and then gradually work and condense it all over the surface of the cavity, so as to get a thin layer as a lining. When this is done the remainder may be almost thrown in with a spade, and the plugs are far more perfect than can possibly be obtained in any other way." At another time he says, "With regard to inserting amalgams, the best way is to pack dry as possible, and to burnish or mallet surface with layer of tin over it to absorb the mercury which rises to surface. If this be once properly tried, there will be no need of trouble either about speroidal tendency or any other method of filling."—*The Dental Record.*

